# Obesity in post menopausal women with a family history of breast cancer: prevalence and risk awareness

**DOI:** 10.1186/1477-7800-6-1

**Published:** 2009-01-08

**Authors:** Parvin Begum, Caroline E Richardson, Amtul R Carmichael

**Affiliations:** 1 Department of Surgery, Russells Hall Hospital, Dudley, West Midlands, DY1 2HQ, UK

## Abstract

**Background:**

Obesity and physical activity are modifiable risk factors in the development of post-menopausal breast cancer. The aim of this study was to assess the level of awareness and prevalence of these factors in women attending family history clinics.

**Methods:**

Women attending the breast cancer family history clinic from 2004 to 2006 completed a questionnaire (SP15 format) about their knowledge of and exposure to various diet and lifestyle factors. All women had been counselled by a Consultant Cancer Geneticist and were given verbal and written information on the effect of life style on breast cancer risk. Responses were analysed using SPSS™ software.

**Results:**

The response rate was 70% and two thirds of women were post-menopausal. The prevalence of obesity in post-menopausal women was 37% with 40% being overweight. The majority of women consumed a healthy balanced diet. Only 15% of post-menopausal women exercised for more than 4 hours per week. Two-thirds of women correctly stated that obesity increases their breast cancer risk and 73% of these were overweight or obese. Over 87% were correctly aware of the role of family history, 68% of a high fat diet, and 57% of hormone replacement therapy in the development of breast cancer.

**Conclusion:**

Women attending family history clinics lead a high risk lifestyle for the development of breast cancer with high prevalence of obesity and low levels of physical activity. A campaign of patient education is needed to promote healthy lifestyle choices, especially physical activity, in these high-risk women.

## Background

Breast cancer is the most common cancer in women in the UK and is twice as frequent as any other female cancer [1]. Women with a family history of breast cancer have a higher risk of developing the disease than the general population. Hill et al estimated between 6% and 19% of women with breast cancer will have a family history of the disease [2] with estimates that up to 27% of women may have an inherited predisposition. Increasing age is a known risk factor and therefore post-menopausal women with a family history of breast cancer are a particularly vulnerable group [1]. Lack of physical activity and obesity are also independent risk factors for developing breast cancer and the increased risk of developing post-menopausal breast cancer attributable to obesity is comparable to that of family history [3]. A 1-point gain in body mass index (BMI) is estimated to increase the risk of postmenopausal breast cancer by 3% and every 5-kg increase in weight increases the relative risk (RR) of developing breast cancer in postmenopausal women by 1.08 [4]. Unfortunately health promotion campaigns have so far failed to highlight this risk. Almost a quarter of the female population in England and Wales were found to be obese in 2006 according to the Annual Health Survey for England [5]. Physical activity has been shown to reduce the risk of developing hormone-receptor positive breast cancer independent of weight reduction [6]. It is imperative to target lifestyle factors in risk reduction for the development of breast cancer in women at high risk of developing breast cancer due to a family history of the disease. Most units specialising in the genetics of breast cancer offer lifestyle advice, but there is no data to show the effectiveness of this prevention strategy.

The aim of this study was to assess the level of awareness of and prevalence of life-style related risk factors, such as obesity, physical activity and high fat diet, in women attending breast cancer family history clinics.

## Patients and methods

This cross-sectional survey was carried out at a large teaching hospital in the West Midlands, England. All women who had attended the Breast Cancer Family History clinics between 2004 and 2006 were invited to participate in order to allow comparison between pre- and post-menopausal women. The questionnaires were in SP15 format.

The patients were handed questionnaires at the family history clinic (FHC) visit or the questionnaires were sent to them through the post. Data on height and weight were used to calculate BMI. The women were questioned on their lifestyle choices such as smoking habits (cigarettes per day), alcohol intake (units per week), level of physical activity (hours per week) and dietary habits. Questions were also asked about their knowledge of conventional risk factors such as reproductive, contraceptive and hormone replacement therapy. For the purpose of this study, post-menopausal status was defined as 50 years of age or above. Women who had previously had a hysterectomy were categorised as post-menopausal.

Responses were analysed with frequencies, single variable and multiple response analyses using SPSS software programme™.

## Results

Of the 130 women who were approached, 92 returned their questionnaire (response rate 71%), although not all women answered all questions and two questionnaires were excluded due to lack of data.

### Demographics

The mode age group was over 60 years (26%), whilst 6% women were 35 years or younger. Seventy-two women responded to the question on ethnicity and the majority (93%), described their ethnicity as white, British, with the remaining 5 describing themselves as Afrocaribbean. Of the 90 women who responded to the question on menopausal status, sixty were post-menopausal.

### Prevalence of obesity

The prevalence of obesity among the post-menopausal women was 36% (21/59) with a further 41% (24/59) in the overweight range. In the pre-menopausal group, less than half of the women (14/29) were categorised as overweight or obese.

### Lifestyle factors

Eighty-eight women stated their alcohol consumption and all but one of the post-menopausal women drank within recommended national limits. Seven of the nine women who smoked were post-menopausal and all nine women stated that they thought smoking increased their risk of breast cancer.

### Dietary intake

**Figure 1:**
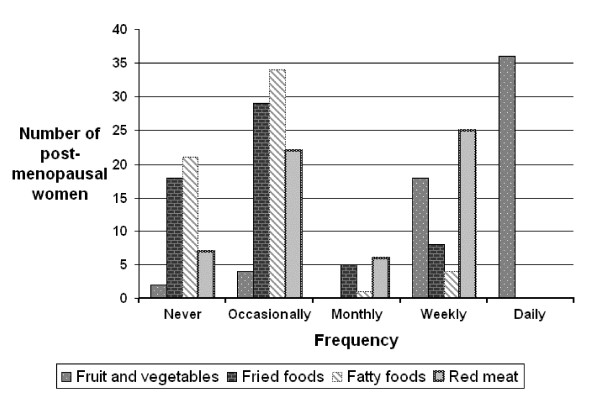
**Dietary intake in post-menopausal women**.

Eighty-nine women answered these questions. Most women (60%) met the Department of Health guideline of eating five portions of fruit and vegetables each day, whilst over a quarter (30%) stated that they never ate any fried food and over half (58%) that they occasionally did. Sixty two percent of women stated that they "occasionally" ate fast foods and 42% ate red meat on a weekly basis. The results for the women in the post-menopausal group are shown in figure 1.

### Physical activity levels

**Figure 2:**
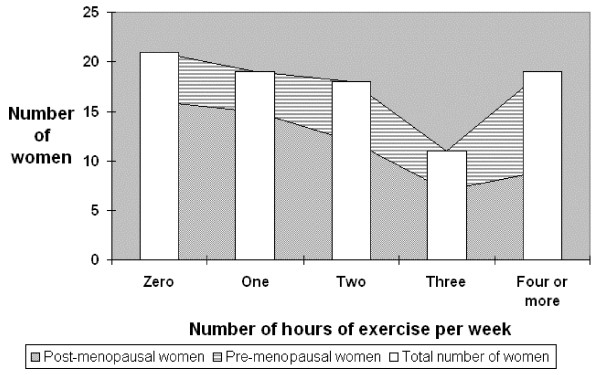
**The number of hours of exercise performed on average per week by pre-menopausal, post-menopausal and all women**.

Eighty-eight women responded to questions relating to their exercise levels. Nearly a quarter (21/88) of all women stated they did no exercise at all and over three-quarters (16/21) of these women were post-menopausal. Only 15% (9/59) of the post-menopausal women compared to 35% (10/29) of pre-menopausal women exercised for more than 4 hours per week (figure 2).

### Knowledge of Risk Factors for the development of Breast Cancer

Family history (91%) and smoking (86%) were the strongest risk factors thought to increase the risk of developing breast cancer. Sixty-seven per cent of post-menopausal women correctly stated that obesity could increase their risk of developing the disease. However, 73% of women who recognised that obesity is a risk factor for developing breast cancer were either overweight or obese. Seventy-three per cent of pre-menopausal women incorrectly stated that obesity increased their individual risk of developing breast cancer.

## Discussion

There is a high prevalence of obesity and being overweight amongst high-risk women attending breast cancer family history clinics. This is particularly the case for post-menopausal women in whom obesity is a risk factor for the development of the disease. The prevalence of obesity in the study population was fairly representative of the UK population, as statistics released by the WHO in 2006 stated 30–40% of women in the post-menopausal age group in the UK were overweight and 25–31% were obese [7]. In the study population, obesity was less well recognised as a risk factor than non-modifiable ones such as family history, or modifiable ones, such as smoking. The majority of women who thought obesity could increase their risk were either overweight or obese. There is a lack of correlation between knowledge of obesity as a risk factor and actual prevalence rates, which may be due to responder bias. This is supported by the fact that many of the pre-menopausal group also marked 'increase' when asked how they thought obesity affected their risk of developing breast cancer. The high percentage of women stating obesity as a risk factor may be due to the recognition of obesity as generally undesirable on health grounds rather than actual knowledge of its effect on the risk of developing breast cancer. Given the high levels of anxiety about developing breast cancer in women attending the family history clinics, it is possible that women may be more likely to maintain a healthy weight if they had formal education linking obesity with the development of breast cancer.

Ecological studies have shown national per capita fat consumption correlates highly with breast cancer mortality rates [8]. Most respondents in the present study only occasionally ate fried food or fast food, ate red meat every week, stated a low level of alcohol intake and most reported eating five portions of fruit and vegetables on a daily basis. Despite their apparent healthy diets, many post-menopausal women still had high BMIs, which is most likely to reflect their very low levels of physical activity. Physical activity has been associated with a reduced risk of between 20 and 40% in the development of breast cancer in both pre-menopausal and post-menopausal women [9]. Increasing the levels of physical activity appears to be the primary target in reducing obesity and subsequent breast cancer risk.

Evidence suggests that lowering a woman's lifetime exposure to oestrogens may reduce the risk developing breast cancer. The only practical strategy in achieving this is through changes in lifestyle, which aim to reduce obesity after the menopause. Post-menopausal women, particularly those in higher risk groups, should be educated on the benefits of maintaining a healthy body weight (BMI between 18.5 and 24.9 kg/m^2^) and actively encouraged and supported to achieve this.

Most women recognised that a positive family history was a risk factor for development of the disease. The reason this figure was not 100% could be due to a lack of awareness or incorrect response ticked on the part of the patient, or inadequate explanation on the part of healthcare professionals. The high level of knowledge of smoking as a risk factor for cancer in general can be credited to the recent campaigns to raise public awareness and could explain the low rates of smoking in these women. This highlights the potential benefit that interventions may have if women are actively educated about the contribution of obesity in the development of post-menopausal breast cancer. Most women attending the family history clinics identify themselves as being at high risk and may be more amenable to engage in health promoting behaviours that are perceived to be risk reducing in nature. Evidence from studies suggests that reducing levels of obesity can reduce the number of breast cancer cases by one tenth [10]. The resultant reduction in mortality makes a public health campaign targeting this area a valuable one.

## Conclusion

Obesity is a potentially modifiable risk factor in the development of breast cancer in post-menopausal women. The prevalence of obesity and being overweight in postmenopausal women with a positive family history are high. There is still a degree of uncertainty amongst women as to the role of obesity in the risk of developing breast cancer. Lack of physical activity appears to be a key area for intervention. This study identifies that there is scope for patient education and weight management by increased physical activity in women attending the family history clinics for breast cancer. Increased physical activity has the potential to decrease breast cancer in post-menopausal women. A campaign of patient education is particularly needed to promote healthy lifestyle choices in this group of patients already at a higher risk of breast cancer due to a positive family history.

## Competing interests

The authors declare that they have no competing interests.

## Authors' contributions

ARC conceived the idea and supervised PB to design the study and statistical analyses. CR participated in writing up the manuscript. All authors read and approved the final manuscript.

## References

[B1] Lahmann PH, Hoffmann K, Allen N, van Gils CH, Khaw KT, Tehard B, Berrino F, Tjonneland A, Bigaard J, Olsen A, Overvad K, Clavel-Chapelon F, Nagel G, Boeing H, Trichopoulos D, Economou G, Bellos G, Palli D, Tumino R, Panico S, Sacerdote C, Krogh V, Peeters PH, Bueno-de-Mesquita HB, Lund E, Ardanaz E, Amiano P, Pera G, Quirós JR, Martínez C, Tormo MJ, Wirfält E, Berglund G, Hallmans G, Key TJ, Reeves G, Bingham S, Norat T, Biessy C, Kaaks R, Riboli E (2004). Body size and breast cancer risk: findings from the European Prospective Investigation into Cancer And Nutrition (EPIC). Int J Cancer.

[B2] Hill AD, Doyle JM, McDermott EW, O'Higgins NJ (1997). Hereditary breast cancer. Br J Surg.

[B3] Hunter DJ, Willett WC (1993). Diet, body size, and breast cancer. Epidemiol Rev.

[B4] Lahmann PH, Schulz M, Hoffmann K, Boeing H, Tjønneland A, Olsen A, Overvad K, Key TJ, Allen NE, Khaw KT, Bingham S, Berglund G, Wirfält E, Berrino F, Krogh V, Trichopoulou A, Lagiou P, Trichopoulos D, Kaaks R, Riboli E (2005). Long-term weight change and breast cancer risk: the European prospective investigation into cancer and nutrition (EPIC). Br J Cancer.

[B5] Health Survey for England Latest Trends 2006.

[B6] Enger SM, Ross RK, Paganini-Hill A, Carpenter CL, Bernstein L (2000). Body size, physical activity, and breast cancer hormone receptor status: results from two case-control studies. Cancer Epidemiol Biomarkers Prev.

[B7] WHO global infobase. http://www.who.int/infobase/report.aspx?rid=118&iso=GBR&Def_Code=cd.

[B8] Armstrong B, Doll R (1975). Environmental factors and cancer incidence and mortality in different countries, with special reference to dietary practices. Int J Cancer.

[B9] Bianchini F, Kaaks R, Vaino H (2002). Weight control and physical activity in cancer prevention. Obes Rev.

[B10] La Vecchia C (1989). Nutritional factors and cancers of the breast, endometrium and ovary. Eur J Cancer Clin Oncol.

